# Machine learning models effectively distinguish attention-deficit/hyperactivity disorder using event-related potentials

**DOI:** 10.1007/s11571-021-09746-2

**Published:** 2022-02-15

**Authors:** Elham Ghasemi, Mansour Ebrahimi, Esmaeil Ebrahimie

**Affiliations:** 1 Institute of Biotechnology, Shiraz University, Shiraz, Iran; 2 School of Animal and Veterinary Sciences, The University of Adelaide, Adelaide, SA 5371 Australia; 3 Department of Biology, School of Basic Sciences, University of Qom, Qom, Iran; 4 Genomics Research Platform, School of Agriculture, Biomedicine and Environment, La Trobe University, Melbourne, VIC 3086 Australia; 5 School of Information Technology and Mathematical Sciences, Division of Information Technology Engineering and Environment, University of South Australia, Adelaide, SA 5095 Australia; 6 School of BioSciences, The University of Melbourne, Melbourne, VIC 3010 Australia

**Keywords:** Attention deficit hyperactivity disorder, Machine learning, Event-related potentials, Frequency bands, Classification, Band power

## Abstract

Accurate diagnosis of Attention-Deficit/Hyperactivity Disorder (ADHD) is a significant challenge. Misdiagnosis has significant negative medical side effects. Due to the complex nature of this disorder, there is no computational expert system for diagnosis. Recently, automatic diagnosis of ADHD by machine learning analysis of brain signals has received an increased attention. This paper aimed to achieve an accurate model to discriminate between ADHD patients and healthy controls by pattern discovery. Event-Related Potentials (ERP) data were collected from ADHD patients and healthy controls. After pre-processing, ERP signals were decomposed and features were calculated for different frequency bands. The classification was carried out based on each feature using seven machine learning algorithms. Important features were then selected and combined. To find specific patterns for each model, the classification was repeated using the proposed patterns. Results indicated that the combination of complementary features can significantly improve the performance of the predictive models. The newly developed features, defined based on band power, were able to provide the best classification using the Generalized Linear Model, Logistic Regression, and Deep Learning with the average accuracy and Receiver operating characteristic curve > %99.85 and > 0.999, respectively. High and low frequencies (Beta, Delta) performed better than the mid, frequencies in the discrimination of ADHD from control. Altogether, this study developed a machine learning expert system that minimises misdiagnosis of ADHD and is beneficial for the evaluation of treatment efficacy.

**Graphical abstract:**

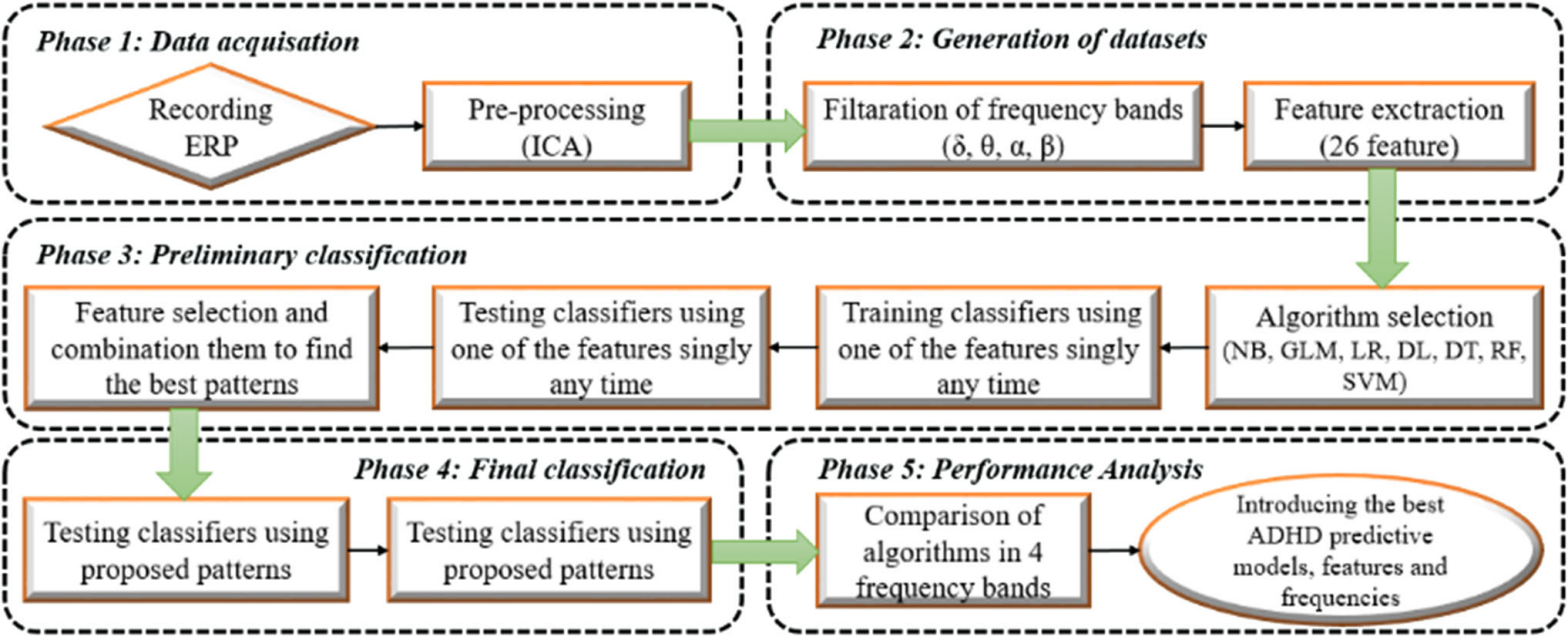

## Introduction

Attention-deficit/hyperactivity disorder (ADHD) is the most commonly diagnosed mental disorder of children with a worldwide prevalence of 7.2% (Thomas et al. 2015). ADHD can remain a disorder throughout the lifetime and is the risk factor for a wide range of other mental health problems including defiant, disruptive, and antisocial behaviors, emotional problems, and self-harm (Willoughby 2003; Shaw et al. 2012). The main symptoms of ADHD in childhood are age-inappropriate inattention, hyperactivity, and impulsivity, which can significantly impact many aspects of behaviour as well as performance, both at school and home (Faraone et al. 2003). At the present, diagnosis of ADHD is often based on a comprehensive assessment by a pediatrician, psychiatrist, or psychologist, but clinical manifestations are not easy to detect. Methods such as magnetic resonance imaging (MRI), Positron Emission Tomography (PET), or Computed Tomography (CT) scan are not yet able to accurately diagnose ADHD, and even in developed countries, the diagnosis of this disorder is controversial among specialists. The risk for false-positive diagnosis is higher in children and diagnoses may be incorrect (Arruda et al. 2019; Ford-Jones 2015). In the case of misdiagnosis, incorrect medications and interventions make the situation worse. Children are given stimulants to increase the frequency, while no parent likes to give their child a medication that they do not need. These drugs are associated with side effects such as loss of appetite, high blood pressure, heart problems, and mood disorders (Volkow and Swanson 2013).

The brain system of humans can be envisioned as a large and complicated network that effectively controls the whole body. The neural tissue of the brain displays anatomical development from childhood to adolescence which is accompanied by changes in oscillatory patterns and brain imaging data, as measured using both EEG and Functional Magnetic Resonance Imaging (*f*MRI) (Smit et al. 2012, 2016; Power et al. 2010), which can also be compared in patients and healthy people. The brain network of people with ADHD has many abnormalities and differences from the brain network of healthy people, and *f*MRI evaluations have revealed those developmental disturbances (Tang et al. 2018). However, their validity is low for ADHD detection while non-imaging data is more important in this regard (Riaz et al. 2018). Abnormal amplitude in ADHD brain waves have been reported by many EEG studies (Ghaderi et al. 2017; Kamida et al. 2016; Li et al. 2018a). ADHD patients have various EEG characteristics that reveal underlying neuropsychological deviation in contrast to other normal people which can be discriminated using machine learning algorithms which are considered as a method for working with complicated data (Chen et al. 2019a; Jamali et al. 2016; Sethu and Vyas 2020). Also, EEG signals, compared with other biometrics, have significant inherent advantages such as universality, uniqueness, cheapness, and Accessibility (Chuang et al. 2013; Sohankar et al. 2015), so that, EEG units can be flexibly deployed in the field including educational and medical institutions.

In general, automated detection based on EEG/ERP signals includes the two main tasks of feature extraction and classification. First studies based on EEG/ERPs to classify patients with ADHD and healthy were published in 2010 and 2011 in which classification accuracies were achieved 90% using SVM (Mueller et al. 2011, 2010). Nazhvani et al. in 2013, reported a classification accuracy of 92.85% in a study where the data were analyzed using nonlinear machine learning (Nazhvani et al. 2013). Most studies have used supervised learning for automatic detection approach. However, the choice of a suitable strategy for machine learning is difficult and thus numerous classification strategies have been developed.

In a recent study, Muller et al. used five classification models comprised of LR, SVM (linear kernel), SVM (radial basis function kernel), RF, and, XGBoost. The models exhibited sensitivities between 75 and 83% and specificities between 71 to 77%. The used features in this study were eyes-closed, eyes-open, and Visual Continuous Performance Test (VCPT) signal power in a series of different frequency bands, as well as ERP Peak Amplitudes and latencies (Müller et al. 2019). It seems that One of the reasons for low efficiency of ADHD detection is inappropriate feature selection for the models. One of the most common EEG characteristics associated with ADHD is power increase at low frequency (Delta, Theta) and/or power decrease at high frequency (Beta) which are occasionally combined and quantified via Theta/Beta Ratio (TBR) (Barry et al. 2003; Arns et al. 2013; Lenartowicz and Loo 2014). An EEG-based adjuvant assessment that uses the TBPR to classify the brain signals of healthy and ADHD subjects has been approved by the Food and Drug Administration (FDA) (Saad et al. 2018). However, some studies suggest that the TBR should be used, while other studies strongly oppose those suggestions (Buyck and Wiersema 2014; Liechti et al. 2013; Loo and Arns 2015). For example, Ogrim et al. found that neither the TBR at Cz nor the Theta and the Beta bands separately were significantly different between patients and controls (Ogrim et al. 2012). Therefore, novel EEG features for ADHD detection are needed.

In a study, the k-Nearest Neighbors (KNN) classifier was applied to Autoregressive (AR) parameters extracted from EEG recorded in attention activity. The accuracy of this supervised learning model was obtained between 85 and 95% (Marcano et al. 2016). In another study (Markovska-Simoska and Pop-Jordanova 2017), to distinguish ADHD patients based on EEG parameters, the highest level of accuracy (99.2%) was obtained using Absolute Band Power in the Theta band at Cz and no acceptable result was obtained at other frequencies and locations. EEG/ERP acquisition has been received at resting-state in most studies, but due to the nature of the ADHD, the EEG/ERP must be recorded in non-restful mode. In this study, the ERP will be recorded at both visual and auditory stimulation. Although some studies have obtained good results, the problem of all studies is that only one or two features are used for the models And algorithms were not optimum or used features were not sufficient for a good classification and stable result. Thus, for a valid diagnosis, the information from EEG may need to be derived via multiple methods. The main objective in this study is to conduct a comprehensive analysis and compare seven machine learning algorithms utilising the decomposed frequency features, previously-used features, and new calculated features, in four different frequencies and finally, introducing of the best prediction models with the highest accuracy and the lowest error rate for a stable and credible diagnosis of ADHD patients.

## Methods

### Participants

The experimental group consisted of 60 children (age between 4 and 15 years) belonging to educational institutes in the Manizales area with written consent from all their parents. 30 of them were ADHD subjects and 30 were in the control group that was diagnosed based on the clinical criteria of the Diagnostic and Statistical Manual of Mental Disorder (DSM-IV) (Stoica and Moses 1997). Patients with pharmacologic management (methylphenidate, 20 mg) did not take the drug until 24 h before the test. All subjects had normal visual and auditory ability, no other neurological disorders and the intelligence levels of both groups were within normal limits (IQ range > 80).

### ERP acquisition

ERP signals were recorded according to the criteria of the Oddball paradigm in two modes of auditory and visual stimulation. In the auditory stimulation state, the subjects were seated in a comfortable chair then the emission of 80 dB tone with a frequency of 1.000 Hz frequent stimulus and 3.000 Hz for infrequent stimulus was presented randomly at every 1.5 s. In visual mode, subjects were asked to stare at a monitor one meter away, which showed a checkerboard image with a consistent pattern as a frequent stimulus. For rare stimuli, a target was presented in the center of the screen with the same pattern in the background. The test consisted of 200 stimuli, of which 80% were non-target stimuli and the remaining 20% were target stimuli. In both test modes, individuals were asked to press a button while presenting an unusual stimulus. The recording was performed using electrodes located in the midline of the head (Pz, Cz, and, Fz) according to a 10–20 international system at a sampling rate of 640 samples per second. Each lead position was considered as stand-alone data and so, the total number of data increased to 6 samples per person. ERP data were recorded using NicoletOne EEG System (VIASYS Healthcare, USA).

### Pre-processing of dataset

Raw ERP data is generally intertwined with various artifacts such as eye movements, blinking, muscle artifacts, and, electrical noise. By considering a threshold of ± 100 μv, a part of these artifacts were excluded from the data. Residual artifacts that could not be removed by filtering due to overlap with the original signals, were removed with the Independent Component Analysis (ICA) automatically. by eliminating dependencies using ICA, space is created where the compounds are independent and the generalization of features is improved. Finally, The cleaned data were transformed from the time domain into the frequency domain using fast Fourier transformation with a 1-s Hanning window and 50% overlap. This analysis was performed with MATLAB software (version 2016).

### Filtration of frequency bands

To better reflect brain activity, the ERP signals were filtered using bandpass filters (elliptic order six) and were decomposed to successive four frequency bands: Beta (13–30 Hz), Alpha (8–12 Hz), Theta (4–8 Hz), and, Delta (< 4 Hz).

### Feature extraction

One of the basic requirements of machine learning and model training is discriminant features. The features that were used are described as follows. MATLAB software (version 2016) was used to calculate the features.

#### Discrete wavelet transform

Eleven features were calculated based on Discrete Wavelet Transform (DWT). DWT is used as an efficient tool to display a signal. By DWT, a signal is decomposed into two levels of the low and the high frequencies called Approximations and Details respectively. Therefore, Wavelet Transform Approximation Coefficients (WT-ApCo) and Wavelet Transform Detail Coefficients (WT-DeCo) were calculated based on a previous study (Yong et al. 1996). Then Approximations Entropy (ApEn) and Details Entropy (DeEn) were calculated. Based on the entropies, the new features were computed including Total Wavelet Entropy (Total WE) that is the total of Detail and Approximation entropies, Relative Approximations Entropy (R-ApEn) that is Approximations Entropy relative to Total Wavelet Entropy, Relative Details Entropy (R-DeEn) that is Details Entropy relative to Total Wavelet Entropy, Approximations Entropy that is normalized with an average of DeEn and ApEn (ApEn-0), Details Entropy that is normalized with an average of DeEn and ApEn (DeEn-0), Approximations Entropy that is normalized with maximum Entropy (ApEn-1) and Details Entropy that is normalized with maximum Entropy (DeEn-1).

#### Band power

The most common parameter used in studies of ADHD has been the estimation of absolute and relative band power. Here, to calculated Absolute Band Power (ABP) according to a study by Hammond et al. (2011), after the raw ERP signal is filtered through bandpass filters to represent the ERP content in the successive frequency bands, in the output of each bandpass filter, each sample first was squared and then their average was computed. Relative Band Power (RBP) is also represented by the percentage of the amplitude in a given frequency band compared with the total amplitude across all frequency bands. New features were also created including Absolute Band Power that is normalized by maximum power (ABP-0), Absolute Band Power that is normalized by the average of powers across all frequencies (ABP-1), Relative Band Power that is normalized by maximum of row ERP power(RBP-0) and Relative Band Power that is normalized by the average of row power (RBP-1).

#### Fractal dimension

Fractal Dimension (FD) is an appropriate tool to analyze EEG/ERP signals and provides a complexity index that describes how the measure of the length of a curve changes depending on the scale. There are several algorithms to calculate FD. Here, the method of the previous paper was used (Jahanshahloo et al. 2017).

#### Autoregressive

Autoregressive (AR) is used to describe a time series. This feature estimates each sample as a weighted sum of previous samples by a recursive linear filter. Here, the standard AR method was used (Moretti et al. 2003), which can model the whole variety of a signal, but they are sensitive to additive noise.

#### Peak amplitude

The Positive Peak Amplitude (PPA), the difference between the positive signal peak and the mid-point of the signal, and the Negative Peak Amplitude (NPA), the difference between the negative signal peak and the mid-point of the signal, were used as two features that are the maximum and minimum value of the ERP signal respectively.

#### Other features

Sum of the ERP signal values (Sum), Average of the ERP signal values (Average), Median of the ERP signal values (Median) also were considered separately for each frequency band. Also, Gender and, First Child were used as complementary features.

### Model selection

To classify patients with ADHD and healthy groups, seven machine learning algorithms described below were used. The ultimate goal was to achieve algorithms that could achieve the highest accuracy in classification with the least number of features. In this study, Rapidminer studio software (version 9.4) was used for designing the models.

#### Support vector machine (SVM)

SVM is a supervised machine learning algorithm for binary classification problems that separate data points in high dimensional space into two classes with a hyperplane that maximizes the gap (so-called ‘margin’) between the hyperplane and support vectors. The standard SVM is restricted to linearly separable data (Mueller et al. 2010), but, sometimes some sets are not linearly separable in two-dimensional space and thus, SVMs can efficiently perform a non-linear classification using what is called the kernel method (Hofmann et al. 2008). Here, dot kernel, the inner product of x and y, were used which is defined as follows; f(x,y) = x*y.

#### Deep learning (DL)

DL is a method based on Artificial Neural Networks (ANN) that consist of multiple layers and neurons that are hidden. The function of these layers is to activate and rectify. This algorithm is trained using back-propagation with random gradient descent as it applies a nonlinear change to its input in the hierarchy and uses what it learns to create a statistical model as output. This continues until the outputs reach a high accuracy (Deng and Yu 2014). A 50-layer network of neurons with a rectifier linear unit was used that selects the maximum of (0, x) where x is the entry value (Ebrahimi et al. 2019).

#### Generalized linear model (GLM)

GLM develops the concept of a standard linear regressions model and allows for response variables that have error distribution models. The GLM model is determined by three components (Breslow 1996), including a random component *f* for the dependent variable *y* (an exponential family of probability distributions), a systematic component (linear model) *η: η* = Xβ and a link function *g* such that E(*y*) = *η* = *g* − 1 (*η*). The result of each dependent variable can be generated from the normal, binomial, Poisson, and, gamma distributions. Here, as regards this dataset is binary, binomial distribution with a 0.5 threshold value was used to classification.

#### Logistic regression (LR)

LR is a simplified version of the GLM operator based on the concept of probability that uses the independent variable (x) to determine the dependent variable (y). In binary regression, input values (x) have two classes (0 or 1) (Rausch and Zehetleitner 2017). Input values (x) are combined linearly using coefficient values or weights (refers to the Greek β) to determine an output value (y). The algorithm builds a regression model to predicts the probability that input data belongs to the labeled class as “0” or “1”.

#### Decision tree (DT)

The DT model uses a tree structure and maps observations of a problem to conclude the value of the target (Che et al. 2011). The technique of this model is that segments a dataset recursively with an in-depth approach until all data items are in a specific class. The structure of a DT model is made of the root, internal nodes, and, terminal nodes (leaf). In this flowchart, the uppermost node is the root, the internal node indicates a test situation on a feature, each branch demonstrates the result of the test condition, and, each leaf node (or terminal node) is specified with a class label. Tree building is performed in top-down with a divide and conquers approach in two steps: tree building and tree pruning (Jadhav and Channe 2016). The construction of new nodes continues until it reaches the stop criteria. The class label prediction is determined based on the majority of samples that have reached this leaf during production and the numerical value is estimated by averaging the values in a leaf. Tree pruning is done in a bottom-up manner to improve prediction and classification accuracy (Ebrahimie et al. 2018). For uniformity and breadth of feature values, the maximal depth 20 and gain-ratio criterion, a kind of information gain, was adjusted for each attribute as previously (Sharifi et al. 2018).

#### Random forest (RF)

RF is a model made up of many individual decision trees. Each tree spits out a class prediction and the class with the most votes considered to be the model’s predictor. Trees are created from the input data set after the sub-sets are formed. Each node represents a division rule for each feature and optimally separates the values based on the selected parameters (Belgiu and Drăguţ 2016). In this study, the number of trees was fixed on 100, maximal depths 10, and, the gain_ratio criterion was also used.

#### Naïve Bayes (NB)

NB is a low-variance classifier based on the Bayes theorem. In the NB model, according to the given value class, the value of each feature is considered independently of the values of the other features (Han et al. 2011). This model has a problem: if in training data the value of a given attribute never occurs in the content of a given class, the probability of the condition will be zero, and when this zero value is multiplied in other probabilities, those values also become zero. To avoid this problem, Laplace correction, adding one to each count of zero values, was used that it has an insignificant effect on the obtained probabilities.

### Preliminary classification

**Fig. 1:**
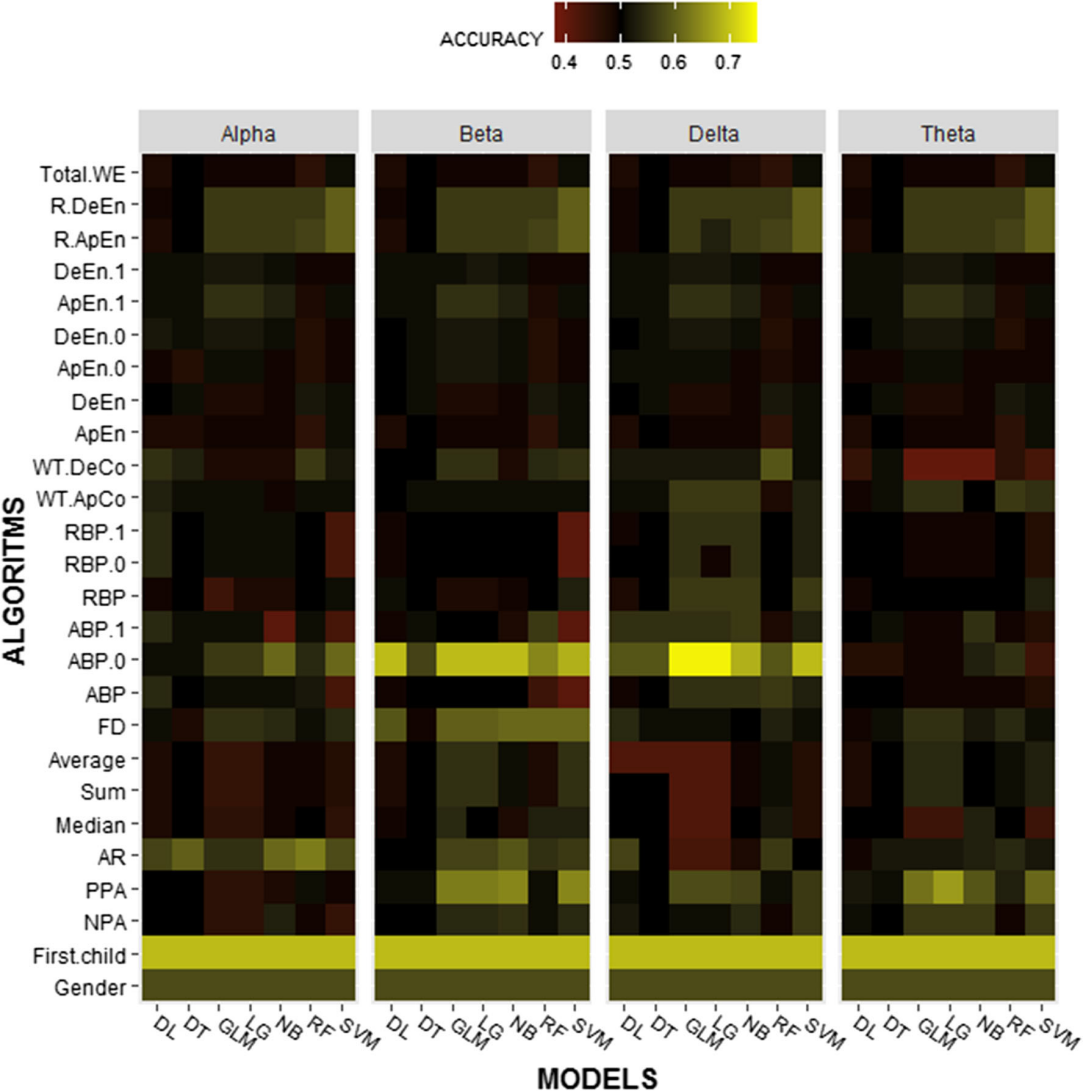
Preliminary classification results for 4 frequency bands; heat map showing the accuracy of the prediction models for Attention Deficit/Hyperactivity Disorder. The color scale indicates the value of the accuracy, the intensity increases from red to yellow. Each column represents one model, and each row represents a feature. In each of the frequency bands, 182 times the execution has been performed, so that at each run, only one of the algorithms and a single feature is used

To limit the number of irrelevant features and achieve the highest accuracy in models, a framework was developed allowing us to select discriminant features and combine them to find the best pattern for each classifier. For this purpose, each model was first trained with only one feature and then tested, and this analysis was repeated for all 26 features in any frequency band distinctly (totally 728 separate analyses). Figure 1 shows the results of these classifications.

### Pattern selection and final classification

As depicted in Fig. 1, for each algorithm in the individual frequency bands some features were better in the criterion of accuracy. A threshold (accuracy > 50) was defined and all of the features that had accuracy higher than 50 were selected for each model distinctly. For example, for the SVM model in the Alpha frequency, the selected features include First-Child, Gender, AR, FD, ABP-0, WT-DeCo, R-ApEn, and R-DeEn as the primary patterns. The process of the pattern selection for all models was done in the same way across four frequencies. In the next step, the classifiers were trained with all of the selected features for each model and were tested. Then, the analysis process was repeated again and again until achieving the best combination of features and highest classification accuracy by removing, adding, and replacing the features.

### Performance evaluation

The performance of the methods was assessed in different ways. To appraise the models built and to improve the predictive potency of the classifiers, all prediction performance measures were estimated using tenfold Cross-Validation (CV) (Kohavi 1995). In CV, all samples are partitioned into K randomly subsets of equal size. This procedure has two nested subprocesses: Training and Testing. Of the K numbers of subset, one subset is kept as a testing dataset and K−1 of the remaining subsets is used to training the model. The cross-validation process is repeated for the total number of subsets, and each subset is used once as testing data. The final estimate is produced by averaging the k results (Rausch and Zehetleitner 2017). In this work, 70% of the data were used for the building of training subsets and 30% of those were used for the testing of models.

The performances of the models were evaluated by the following measures:

The ratio of correctly classified examples or percentage of right predictions is considered as accuracy:

$$Accuracy = {\raise0.7ex\hbox{${\left( {TP + TN} \right)}$} \!\mathord{\left/ {\vphantom {{\left( {TP + TN} \right)} {\left( {TP + TN + FP + FN} \right)}}}\right.\kern-\nulldelimiterspace} \!\lower0.7ex\hbox{${\left( {TP + TN + FP + FN} \right)}$}}.$$

Precision which indicates what proportion of positive identifications was actually correct:

$$Percision = {\raise0.7ex\hbox{${TP}$} \!\mathord{\left/ {\vphantom {{TP} {\left( {TP + FP} \right)}}}\right.\kern-\nulldelimiterspace} \!\lower0.7ex\hbox{${\left( {TP + FP} \right)}$}}.$$

Classification error is calculated as the number of all incorrect predictions divided by the total number of the dataset:

$$Classification error = {\raise0.7ex\hbox{${\left( {FP + FN} \right)}$} \!\mathord{\left/ {\vphantom {{\left( {FP + FN} \right)} {\left( {TP + TN + FP + FN} \right)}}}\right.\kern-\nulldelimiterspace} \!\lower0.7ex\hbox{${\left( {TP + TN + FP + FN} \right)}$}}.$$

Besides, the Receiver Operating Characteristic (ROC) was used to evaluate the performance of models at all classification thresholds. ROC curve for binary classification with different discrimination thresholds is displayed as a graphical layout of true positive rate (sensitivity) versus false positive rate (one minus the specificity):

$$Sensitivity = {\raise0.7ex\hbox{${TP}$} \!\mathord{\left/ {\vphantom {{TP} {TP + FN}}}\right.\kern-\nulldelimiterspace} \!\lower0.7ex\hbox{${TP + FN}$}}$$

$$\left( {1 - Specificity} \right) = {\raise0.7ex\hbox{${FP}$} \!\mathord{\left/ {\vphantom {{FP} {FP + TN}}}\right.\kern-\nulldelimiterspace} \!\lower0.7ex\hbox{${FP + TN}$}}$$

here TP, TN, FP, FN are the number of true positives, true negatives, false positives, and, false negatives, respectively. The Area Under the ROC Curve (AUC) can be applied as a reliable measure of classifier performance because it provides a single measure of overall accuracy that is not dependent on a particular threshold. The maximum value of AUC is 1 that denotes a perfect prediction (Bradley 1997).

## Results

### Feature selection based on the classification

To investigate the contribution of each feature to the predictive model, a different method was used. First, the classification was performed with only one feature. Figure 1 shows the feature importance across different predictive models in Separate frequencies. Features that were very accurate in the classification had to be selected. Several features, such as Total WE, R-DeEn, R-ApEn, Gender, and some others, had the same results in all classifiers, while a few of them had varied results. For example, the GLM, LR, and, SVM had high accuracy using ABP-0 in the Delta frequency. In addition to these three models, DL had high accuracy using this feature in the Beta band. the NB and SVM models had moderate accuracy in the Alpha frequency. In contrast, none of the models did have significant accuracy using ABP-0 in the Theta band (Fig. 1). Although this was a primary classification, it helped a lot in the selection of important patterns to increase the accuracy of the models for the next steps.

### Classification performance assessment

The results of this study showed that the simultaneous use of several right features has a significant role in improving the accuracy of classification. For this purpose, the classifications were performed using the primary patterns based on the important features. Although their accuracy improved compared to when only one feature was used in the models, the results were still not acceptable. So the classification was repeated several times and each time the combination of features was changed, by deleting, adding, and replacing them, to discover a specific pattern for each model that can increase prediction accuracy to the highest level. According to Table 1 in the Delta frequency, GLM, LR, and, DL had the best results using only two features. SVM did not perform well compared to the previous three models, because despite having more number features, it had a classification error (%3.70) and its accuracy did not exceed %96.30. The NB model had the lowest efficiency compared to the others, and its best status was a combination of the three features including Gender, First Child, and ABP-0. No other features were found that could improve the accuracy of this classifier. At the Theta frequency (Table 2) GLM and LR were able to discriminate between ADHD and non-ADHD children with 100% accuracy. In this frequency, one of the main features of GLM, in addition to the features used for it in the Delta band, was WT-ApCo. Although DL had a precision of %100, its accuracy compared with the Delta band decreased and it had a Classification error(%1.85). SVM represented the worst performance in this frequency. As well as at the Alpha frequency (Table 3), three models including GLM, LR, and DL had the highest accuracy and precision using only two features while the Classification error was also zero. After that, SVM had the best performance using five features. But according to the results in Table 4, the three algorithms of GLM, LR, and DL had remarkable results in the Beta band and it seems that they are almost the same in the used patterns and performance in the frequencies of Beta, Alpha, and Delta. The best results for DT were obtained at this frequency (accuracy 97.22). Although the performance results for SVM were the same at both Beta and Alpha frequencies, it was better evaluated at the Beta band in general because it was able to achieve the same performance with fewer features.

**Table 1:** Algorithm performance using the best optimum combination of multiple features for the Delta frequency band dataset

Classifier	Feature(s) used (pattern)	Performance analysis (%)		
		Accuracy	Precision	Prediction error
NB	Gender, First child, ABP-0	78.70	81.36	21.30
GLM	ABP-0, ABP-1 or RBP-0 or RBP-1	**100.00**	100.00	–
LR	ABP-0, ABP-1 or RBP-0 or RBP-1	**100.00**	100.00	–
DL	ABP-0, ABP-1 or RBP-0 or RBP-1	**100.00**	100.00	–
DT	ABP-0, ABP-1	95.37	94.55	4.63
RF	ABP-0, ABP-1	91.67	90.91	8.33
SVM	ABP-0, FD, RBP, WT-ApCo, WT-DeCo, DeEn	96.30	93.10	3.70

Using “or” among features means that the importance of these attributes are the same and can be used instead of each other

Bold indicates models with high accuracy

**Table 2:** Algorithm performance using the best optimum combination of multiple features for the Theta frequency band dataset

Classifier	Feature(s) used (pattern)	Performance analysis (%)		
		Accuracy	Precision	Prediction error
NB	Gender, First child, ABP-0	75.93	81.36	24.07
GLM	ABP-0, WT-ApCo, RBP-0 or RBP-1	**100.00**	100.00	–
LR	ABP-0, RBP-0 or RBP-1	**100.00**	100.00	–
DL	ABP, ABP-0, ABP-1 or RBP-0 or RBP-1	**98.15**	100.00	1.85
DT	Gender, First child, ABP, AR	78.70	94.55	21.30
RF	Gender, First child, ABP, ABP-0, ABP-1, AR	77.78	90.91	22.22
SVM	NPA, PPA, AR, R-ApEn, DeEn, ApEn, ApEn-1	72.22	93.10	27.78

Using “or” among features means that the importance of these attributes are the same and can be used instead of each other

Bold indicates models with high accuracy

**Table 3:** Algorithm performance using the best optimum combination of multiple features for the Alpha frequency band dataset

Classifier	Feature(s) used (pattern)	Performance analysis (%)		
		Accuracy	Precision	Prediction error
NB	Gender, First child, FD, AR, ApEn-1, ABP-0, ABP-1	75.93	75.00	24.07
GLM	ABP-0, RBP-0	**100.00**	100.00	–
LR	ABP-0, RBP-0	**100.00**	100.00	–
DL	ABP-0, RBP-0	**100.00**	100.00	–
DT	ABP, ABP-0, ABP-1	91.67	92.45	8.33
RF	Gender, First child, ABP, ABP-0, RBP-1, RBP-0, WT-DeCo, AR, R-DeEn, R-ApEn	77.78	84.09	22.22
SVM	ABP-0, ABP-1, AR, FD, R-DeEn	**98.15**	100.00	1.85

Bold indicates models with high accuracy

**Table 4:** Algorithm performance using the best optimum combination of multiple features for the Beta frequency band dataset

Classifier	Feature(s) used (pattern)	Performance analysis (%)		
		Accuracy	Precision	Prediction error
NB	Gender, First child, PPA, ABP-0, AR, FD	78.70	81.36	21.30
GLM	ABP-0, RBP-0 or RBP-1	**100.00**	100.00	–
LR	ABP-0, RBP-0 or RBP-1	**100.00**	100.00	–
DL	ABP-0, RBP-0 or RBP-1	**100.00**	100.00	–
DT	ABP-0, ABP-1	**97.22**	96.36	2.78
RF	ABP-0, ABP-1	95.37	92.98	4.63
SVM	ABP-0, ABP-1	**98.15**	100.00	1.85

Using “or” among features means that the importance of these attributes are the same and can be used instead of each other

Bold indicates models with high accuracy

According to Tables 1, 2, 3 and 4 GLM and LR in all four frequency bands were equally and excellently able to distinguish ADHD in children, and frequency did not have much effect on the performance of these algorithms. DL also was well evaluated in all frequencies except the Theta band, which was slightly reduced in performance. DT had better results in the Beta and the Delta bands, respectively than other frequencies. Beta and Alpha bands are appropriate for SVM To some extent, and the Theta frequency is not recommended for classification by SVM at all. Although RF performed relatively well at the Beta frequency and the overall performance of RF was much better than NB, these two models do not seem to be appropriate classifiers for detecting ADHD.

### Contributing features

The high performances achieved by supervised learning are due to the ability of the calculated features. Results showed that if multiple features are used simultaneously, they can increase or decrease the efficiency of the predictive model, therefore the election of features with a Strengthening effect for each other is very substantial. Tables 1, 2, 3 and 4 show specific and optimal patterns for the models. Of the 26 attributes, modified features based on band power including ABP-0, ABP-1, RBP-0, and RBP-1, that were first used in this study, were identified as the best features that could describe ERP signals for ADHD discrimination as well. Among these, ABP-0 was recognized as the most key feature, so that by removing it, the efficiency of the algorithms was greatly decreased and there was no replacement for it, while ABP-1, RBP-0, and, RBP-1 had the almost same value and could be used instead of together.

### Comparison of the efficiency of models

**Fig. 2:**
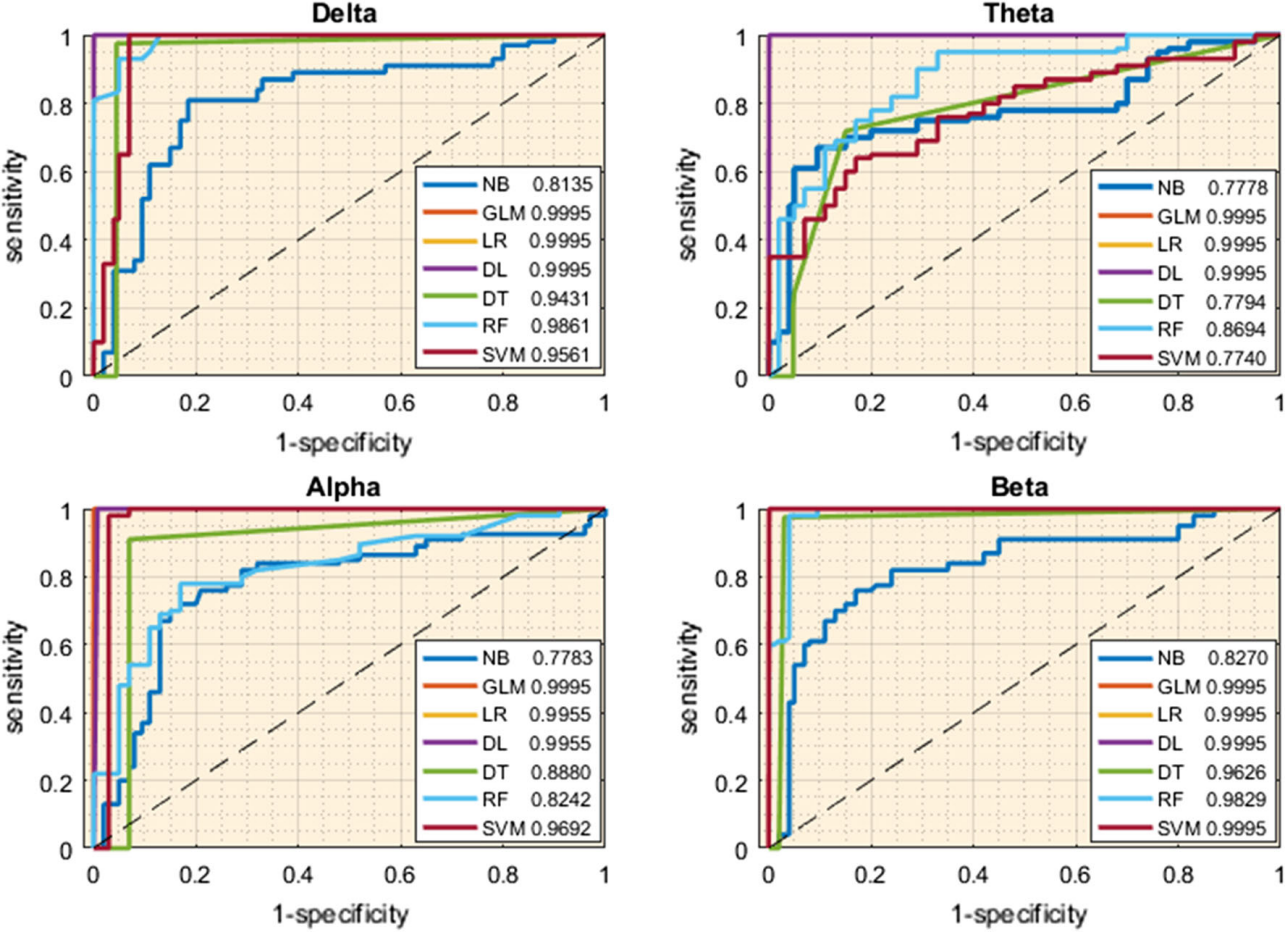
ROC curves obtained from seven machine learning algorithms in four frequency bands when proposed features in Tables 1, 2, 3 and 4 are used. Generalized Linear Model, Logistic Regression, and Deep Learning display a perfect discrimination in all four frequencies

It is important to conduct an evaluation that employs multiple performance indices. Thus, the models were evaluated using other indices including ROC and AUC which are commonly used in machine learning. As described above, to draw a ROC curve, a positive or negative confidence value for each specimen must be provided by the classifier. If the model has the predictive ability, the ROC curve is placed above the diagonal, and conversely, it has no ability to predict. Figure 2 summarizes the ROCs comparisons between the proposed models at different frequencies. All the curves have fallen above the diagonal that means they have all very sensitive. In medical diagnoses, a high true-positive rate is more desirable than a lower false-positive rate. Ideally, when the classifier's ability to predict is 100%, the ROC curve coincides with the y-axis. Also, AUC is commonly used as a natural criterion to describe the performance of the classifier based on the ROC curve. According to Fig. 2, the AUCs values for GLM, LR, DL in all frequencies, and, SVM in the Alpha and Beta bands are equal to 1 and an AUC of 1 is equivalent to perfect discrimination. After them, the maximum of AUCs with a value of 0.99% and 0.98% were for RF in the Delta and Beta bands, respectively. As well as SVM in the Delta frequency and DT in the Beta frequency attained AUC of 0.96 and NB also had no significant results compared to others.

### Discriminative frequencies

**Fig. 3:**
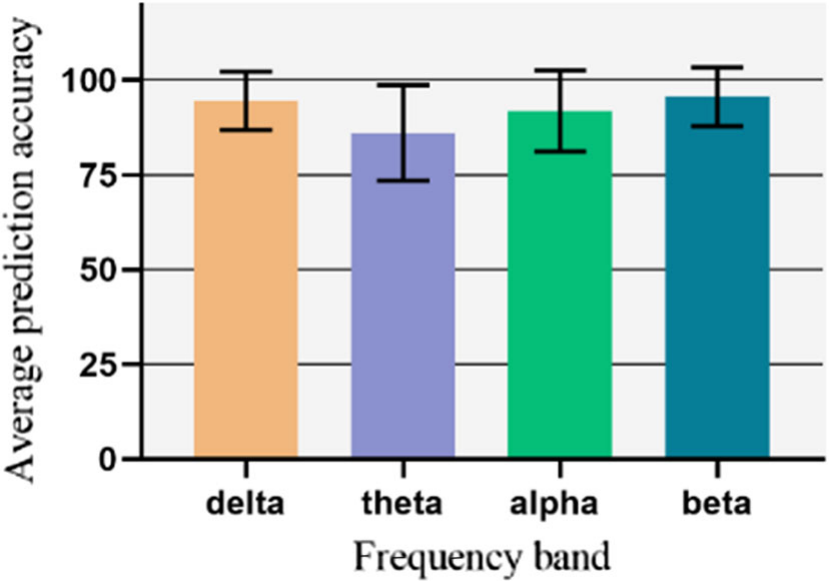
Pediction mean accuracy and Prediction mean error for frequency bands

The results of this study showed that ERP spectral characteristics can be used to identify ADHD children. Seven machine learning models were applied to different frequency bands. The results derived from their average accuracy and classification errors showed that the high frequencies (Beta) and low frequencies (Delta) perform better than the mid frequencies for differentiation of ADHD (Fig. 3). This is a general conclusion and comparison between different frequencies, but for the middle bands, some models have very high efficiency. For the Delta band, This corresponds with previous studies (Öztoprak et al. 2017), but for other frequencies, especially Beta, this is the first great result to be reported.

## Discussion

In this paper, a comprehensive study was conducted that utilized multiple strategies such as Event-Related Potential (ERP), Different levels of brain frequency, Complementary features, and Various algorithms to reveal hidden potentials of ADHD children's brain waves and tried to build classifiers that can effectively detect candidates for ADHD in a machine learning approach. This study can be particularly interesting for various reasons. First, Analysis is based on ERP data with both auditory and visual stimulation, which makes the possibility to follow cognitive processes non-invasively at the millisecond scale. second, opposite to previous researches where one or two frequency bands have been emphasized, In the current findings, it seems that all four frequencies (Delta, Theta, Alpha, Beta) can separately be used to distinguish brain waves in ADHD prediction. Third, new features were defined, and through the innovative method of selection and combination of features, a specific pattern was identified for each model that could increase the efficiency of the model. Finally, With the strategies used, various machine learning algorithms were compared in similar conditions, and the highest accuracy was obtained for each model compared to previous studies.

The focus of this study was on ERP data because the event-related data are more sensitive and provide better performance than spontaneous EEG data recorded while the participants are at a resting state. Various studies have shown that patients with ADHD display brain alterations using ERP attributes (Müller et al. 2019; Lenartowicz and Loo 2014; Öztoprak et al. 2017; Li et al. 2018b; Kaur et al. 2020). However, the pre-processing method may also improve the performance of EEG data. For example, similar studies using the EEG spectrum with successful classification (> 94%) (Pereda et al. 2018; Dea et al. 2019), suggest that different strategies to reduce its dimensionality may affect its classification ability. Therefore, it is important to adopt a data-based approach. Various machine learning techniques were introduced based on the ERP component. Evidence shows that the activity of brain waves can be increased during voluntary behaviour (Karch et al. 2016) and ERP signal is a better criterion for separating these distinctions (Sabeti and Boostani 2020). Vahid et al. (Vahid et al. 2019), used an ERP signal in the range between 0.5 and 20 Hz for DL classification and obtained an accuracy of 83%. In another recent study, Muller et al. (2019), applied an LR and SVM method on event-related data in the domain of 0.5–50 HZ and achieved an accuracy of around 85%. It is well-known that ERP data cannot alone provide sufficient information for discriminating between ADHD and healthy cases. ERPs were decomposed into four frequency bands (< 4 HZ, 4-8HZ, 8-12HZ, 13-30HZ). The finding indicated that features extracted from Separate frequencies are better able to reveal differences in ADHD brain changes. Several studies confirm this (Markovska-Simoska and Pop-Jordanova 2017; Öztoprak et al. 2017; Kiiski et al. 2019; Jouzizadeh et al. 2020; Shephard et al. 2018). In this context, similar studies suggested that certain frequencies were major in ADHD classification. Markovska et al. (Markovska-Simoska and Pop-Jordanova 2017), found that the EEG patterns are following brain Maturational processes, and Delta and Theta bands have discriminator features in ADHD children. A classification analysis showed that Alpha modulation has a high capability to recognize ADHD in children (Guo et al. 2019). Few studies have been performed based on ERP. Ghassemi et al. (Ghassemi et al. 2010), applied a KNN classifier on several frequency features that were extracted from different independent ERP components. They obtained high accuracy (92%) in the Gama band. To identify discrimination frequencies in ADHD approaches, seven machine learning algorithms were applied to the Delta, Theta, Alpha, and, Beta bands. In contrast to previous studies, the result showed that there is no significant difference between frequencies in the average (Fig. 2). However, slow and high frequencies were a little better in comparison to middle frequencies.

The next factor that makes this work more prominent than previous studies is the definition of new features, finding complementary features, and combining them to create specific patterns for each algorithm to building the best discrimination system. To understand the importance of each feature, initially, only one feature was used for the classifiers. For example, to classify the Alpha frequency dataset by DL model, Initially, the classification was performed only with Gender as a discriminate feature and the second time, the classification was performed with First-Child, the third time, the classification was performed with NPA, and so it went on. Therefore, 26 separate classifications were performed for DL in the Alpha band and this was repeated for the other algorithms at each frequency. In this way, the classification accuracy for each feature was obtained in all models (Fig. 1). The highest accuracies were between 70 and 75%. To increase efficiency, the features with an accuracy above 50 were selected and all used together for classifiers. For example, in the Alpha band for DL (first column on the left in Fig. 1), Gender, First-Child, AR, ABP, ABP.1, RBP.0, RBP.1, WT.ApCo, WT.DeCo were selected and again the classification was performed with all of these. It was observed that the efficiency of the models did not increase significantly when several features were used. It seems that increasing complexity causes overfitting and also reduces the generalizability of the model. To resolve this issue, the combination of the features was changed, by removing and replacing them. In the example above, first, Gender was removed and classification was performed with the remaining 8 features. Next time First-Child was removed and gender was added to the combination again. The third time, both were removed from the classification and the efficiency of the model was measured. This was repeated until the highest accuracy was achieved, and finally ABP-0, RBP-0 were obtained as the best discriminatory combination for the DL model at the Alpha frequency (Table 3). This method was repeated to achieve the discriminatory and specific combination of features for each model at each frequency. These results proved that some of the features, when used together, can increase the performances of models much more than when they are used alone, in other words, they have synergistic effects. conversely, some of them, when combined, reduce the efficiency of the model probably due to the increased complexity and inconsistency. Thus, finding complementary features can be forward progress for most machine learning-based methods. The features listed in Tables 1, 2, 3 and 4 were obtained after repeating the classification several times and are introduced as the best combination for each model at different frequencies. Also, the new features that were defined based on band power, including Absolute Band Power-0 (ABP-0), Absolute Band Powe-1 (ABP-1), Relative Band Power-0 (RBP-0), and, Relative Band Power-1 (RBP-1) were identified as the most effective features. These can complement each other's effects and increase the efficiency of the models to the maximum. ABP-1, RBP-0, and RBP-1 were almost of equal value and could sometimes be used instead of each other, but ABP-0 was much more valuable than the others as if by removing it, the accuracy of the models were remarkably reduced and it could not be replaced by any other feature. The importance of power-based features has been confirmed by previous studies (Kamida et al. 2016; Markovska-Simoska and Pop-Jordanova 2017; Tenev et al. 2014; Khoshnoud et al. 2018). However, their performance was significantly improved due to the changes made in this analysis.

In addition to the strategies mentioned above that were used to improve the performance of the models, finding the appropriate algorithm for the data type is the most important factor in the success of artificial intelligence-related tasks. Here we compare seven machine learning models under similar conditions to find out which algorithm is more efficient in distinguishing the decomposed ERP data of healthy and ADHD children. The highest accuracies in this study are related to GLM and LR in all frequencies (100%), DL in the Delta, Beta, and, Alpha bands (100%), DL in the Theta band (98.15%), SVM in the Alpha and Beta bands (98.15%) and DT in the Beta band (97.22%). Accuracy alone may not be a sufficient criterion for validation of the model, so AUC was calculated for all models based on sensitivity and specificity. The requirements for an ideal ADHD marker, as defined by Thome et al. (2012), are diagnostic sensitivity and specificity values > 80%. Thus, the majority of classifiers used are evaluated in optimal to excellent state. In addition, a tenfold cross-validation technique was used to evaluate all models. The LR model is practically a type of GLM which is often used in ADHD machine learning studies. In a cross-sectional analysis, Liechti et al. (Liechti et al. 2013), applied the LR model on the Theta/Beta ratio feature based on EEG, but found only 53% accuracy in ADHD prediction. Recent studies have shown that this feature has not been able to successfully classify ADHD and healthy subjects (Dijk et al. 2020; Kiiski et al. 2020). Recently, Muller et al. (2019), compared two sets of features included peak amplitudes and latencies of independent ERP components, and decomposed ERP using LR. The AUC obtained was 84% and 85% respectively. These results show that although decomposed ERP signal improved performance, it seems that appropriate features have not been used. Our analysis proved that regression-based models can be introduced as the most powerful algorithms in ADHD patients’ diagnosis if the appropriate feature set is used.

Recently, several DL approaches in ADHD detection have been considered. Dubreuil et al. (2020), trained a Convolutional Neural Network (CNN) with a four-layer architecture in combination with a filtering and pooling model using decomposed multi-channel EEG/ERP time–frequency, Independent of manual feature selection, and reported that the event-related spectrograms provide greater accuracy (88%) compared to resting state (66%). In another study, Mohammadi et al. (2016) used Multi-layer perceptron (MLP) to classify EEG signals of 30 healthy and 30 ADHD children using linear features. The use of manual features increased the classification accuracy to 93.64%. In another similar study, Chen et al. (2019b), used three different models include MLP, SVM, and, CNN to classify ADHD and compared their performance. CNN with 94.67% accuracy was better than MLP and SVM models. Although the implementation of various methods can improve the performance of an algorithm, due to the nature of DL, which is basically compatible with big data, these results are generally obtained from small-size data that can not be dependable for diagnostic tasks. In this study, to moderate the size of the dataset, qualified features that are extracted directly from the ERP were used to train the artificial neural network and the number of hidden layers was increased to 50 layers which Improved the complexity and efficiency of the model. Compared with previous DL models, the method used here so far has the best classification result for DL in separate frequencies (accuracy 100% in Delta, Beta, Alpha bands and, accuracy 98.15% in Theta band) which can be suggested for validation with bigger data. The SVM is also a classifier that has been used more for ADHD research. In a study based on ERP data, Oztopark et al. (2017), used a Time–Frequency Hermite-Atomizer (TFHA) technique for the extraction of high-resolution time–frequency domain features and SVM-Recursive Feature Elimination to classify ADHD and healthy groups. They obtained high accuracy (100%) and introduced the Delta band as the most contributing frequency band. In this study, SVM only gained better accuracy (98.15) in the alpha and beta bands. The difference between these results and the findings of this article is due to several factors such as optimizing the algorithm, feature type, and, feature extraction techniques and also confirms this hypothesis that classification performance in each frequency can be improved by optimizing this technique. NB, RF, and, DT were less commonly used to classify brain signals. The highest accuracies for NB and RF were 87% and 83% respectively that were obtained by Altinkaynak et al. (2020). In this paper, NB had the lowest performance compared to other models, and its performance did not improve with the method of this article. It does not seem to be a good model for the classification of the EEG/ERP data. However, DT was first used in this study and it was better on average than RF. These results suggest that in addition to the fact that some algorithms are inherently better at classifying brain signals, the use of different strategies can reduce or improve their performance.

This study has several limitations. First, the dataset used was small. It is better to use more data for supervised learning, especially for deep learning that is inherently compatible with big data. Second, age range between 4 to 15 was evaluated, but, to define a standard protocol, it is necessary to age segregation for children, adolescents, and, adults because, with age, the symptoms of ADHD and brain characteristics also change. Third, the subtypes of ADHD including ADHD-I, ADHD-H, and ADHD-C were not analyzed in this study. Finally, this study was performed on healthy and ADHD subjects, while a computational expert system for ADHD diagnosis should be able to distinguish ADHD from other mental disorders. This requires further investigation with big data.

## Conclusion

In this study, seven machine learning algorithms were examined to achieve the best autonomic model for the diagnosis of ADHD. The findings suggested that multiple factors are involved in determining a successful guideline for classifying the brain signals of ADHD patients and healthy subjects. The high performances of classifiers in the present study are due to the calculated discriminative features, feature selection method, and, certainly, identification of complementary features and combination of them. The developed machine learning pipeline can be used for other diseases with brain signal recordings and the information obtained in this study can be very helpful for developing an expert diagnostic system. Overall, our results provide new insight about accurate machine learning that can minimize misdiagnosis also be used to evaluate the effectiveness of treatment.
